# Adiponectin Haploinsufficiency Promotes Mammary Tumor Development in MMTV-PyVT Mice by Modulation of Phosphatase and Tensin Homolog Activities

**DOI:** 10.1371/journal.pone.0004968

**Published:** 2009-03-25

**Authors:** Janice B. B. Lam, Kim H. M. Chow, Aimin Xu, Karen S. L. Lam, Jing Liu, Nai-Sum Wong, Randall T. Moon, Peter R. Shepherd, Garth J. S. Cooper, Yu Wang

**Affiliations:** 1 Department of Pharmacology and Pharmacy, University of Hong Kong, Hong Kong, China; 2 Department of Medicine, University of Hong Kong, Hong Kong, China; 3 Research Center of Heart, Brain, Hormone, and Healthy Aging, University of Hong Kong, Hong Kong, China; 4 Department of Biochemistry, University of Hong Kong, Hong Kong, China; 5 Open Laboratory of Chemical Biology of the Institute of Molecular Technology for Drug Discovery and Synthesis, University of Hong Kong, Hong Kong, China; 6 Howard Hughes Medical Institute, University of Washington School of Medicine, Seattle, Washington, United States of America; 7 School of Biological Sciences, University of Auckland, Auckland, New Zealand; 8 Maurice Wilkins Centre for Molecular Biodiscovery, University of Auckland, Auckland, New Zealand; Dresden University of Technology, Germany

## Abstract

**Background:**

Adiponectin is an adipokine possessing beneficial effects on obesity-related medical complications. A negative association of adiponectin levels with breast cancer development has been demonstrated. However, the precise role of adiponectin deficiency in mammary carcinogenesis remains elusive.

**Methodology/Principal Findings:**

In the present study, MMTV-polyomavirus middle T antigen (MMTV-PyVT) transgenic mice with reduced adiponectin expressions were established and the stromal effects of adiponectin haploinsufficiency on mammary tumor development evaluated. In mice from both FVB/N and C57BL/6J backgrounds, insufficient adiponectin production promoted mammary tumor onset and development. A distinctive basal-like subtype of tumors, with a more aggressive phenotype, was derived from adiponectin haplodeficient MMTV-PyVT mice. Comparing with those from control MMTV-PyVT mice, the isolated mammary tumor cells showed enhanced tumor progression in re-implanted nude mice, accelerated proliferation in primary cultures, and hyperactivated phosphatidylinositol-3-kinase (PI3K)/Akt/beta-catenin signaling, which at least partly attributed to the decreased phosphatase and tensin homolog (PTEN) activities. Further analysis revealed that PTEN was inactivated by a redox-regulated mechanism. Increased association of PTEN-thioredoxin complexes was detected in tumors derived from mice with reduced adiponectin levels. The activities of thioredoxin (Trx1) and thioredoxin reductase (TrxR1) were significantly elevated, whereas treatment with either curcumin, an irreversible inhibitor of TrxR1, or adiponectin largely attenuated their activities and resulted in the re-activation of PTEN in these tumor cells. Moreover, adiponectin could inhibit TrxR1 promoter-mediated transcription and restore the mRNA expressions of TrxR1.

**Conclusion:**

Adiponectin haploinsufficiency facilitated mammary tumorigenesis by down-regulation of PTEN activity and activation of PI3K/Akt signalling pathway through a mechanism involving Trx1/TrxR1 redox regulations.

## Introduction

The prevalence of obesity and obesity-related cancers has risen alarmingly for the past several decades [1], [2], [3]. Unfortunately, the mechanisms underlying the association between obesity and cancer are not well understood. Recent evidences suggest that adipokines, referring to a group of secreted factors from adipose tissue, could be the key players in regulating obesity-related carcinogenesis [4], [5], [6], [7]. Adiponectin is an abundant adipocyte-derived hormone that can elicit pleiotropic beneficial functions against obesity-related medical conditions, such as diabetes, chronic inflammation, atherosclerosis and tumorigenesis [8], [9]. Decreased circulating concentrations of adiponectin are associated with many obesity-related cancer diseases, including breast cancer, endometrial cancer, gastric cancer, colorectal cancer, renal cell carcinoma and prostate cancer [10], [11], [12], [13], [14], [15], [16]. Breast cancer represents the second leading cause of death among women. An inverse correlation of circulating adiponectin levels with breast cancer risk has been observed in both pre- and post-menopausal women, independent of body mass index and other known risk factors [17], [18], [19], [20], [21], [22], [23], [24]. Moreover, mammary tumors arising in women with low serum adiponectin levels are more likely to show a biologically aggressive and poor prognosis phenotype. These epidemiological evidences suggest that reduced adiponectin expression might be causally involved in obesity-related carcinogenesis.

In line with these clinical findings, numerous experimental evidences support the role of adiponectin as an inhibitory factor for breast cancer development [25], [26], [27], [28], [29], [30], [31], [32], [33]. Adiponectin at physiological concentrations suppresses the proliferation and causes cell cycle arrest in both estrogen receptor (ER)-negative and ER-positive human breast carcinoma cells. It inhibits insulin- and growth factors-stimulated growth of ER-positive breast cancer cells [28]. Adiponectin replenishment suppresses mammary tumorigenesis of MDA-MB-231 cells in nude mice [28]. Cell-type dependent signalling mechanisms have been suggested to mediate the growth inhibitory effects of adiponectin. In MCF-7 cells, adiponectin induces AMP-activated protein kinase (AMPK) phosphorylation and inactivates p42/p44 MAPkinase (ERK1/2) [29]. By contrast, the inhibitory effects of adiponectin on T47D cell growth are associated with inactivation of ERK1/2 but not AMPK or p38 MAPK [18], [28]. In MDA-MB-231 cells with ectopic ER over-expression, globular adiponectin inhibits cell proliferation by blocking JNK2 signalling [26]. In ER-negative MDA-MB-231 cells, adiponectin could modulate the glycogen synthase kinase-3beta (GSK3beta)/beta-catenin signaling pathway [28]. Prolonged treatment with adiponectin markedly reduces serum-induced phosphorylation of GSK3beta, decreases intracellular accumulation and nuclear translocation of beta-catenin, and suppresses cyclin D1 expression. Despite of these progresses, whether adiponectin deficiency is a direct contributor to the pathogenesis of breast cancer remain elusive.

In this study, we investigated the effects of reduced adiponectin expression on mammary tumor development in MMTV-PyVT transgenic mice. Mice with reduced adiponectin expressions were established in both FVB/N and C57BL/6J backgrounds. Adiponectin haploinsufficiency significantly reduced tumor latency and promoted mammary tumor development in both female and male animals. The results demonstrated that inadequate adiponectin production might alter the stromal microenvironment towards more pro-proliferative and pro-tumorigenic in mammary tissue, by triggering the abnormal redox activities that led to the inhibition of tumor suppressor PTEN and hyperactivation of PI3K/Akt signaling pathways in mammary tumor cells.

## Results

### Adiponectin haploinsufficiency promotes mammary tumor development in MMTV-PyVT mice

**Figure 1:**
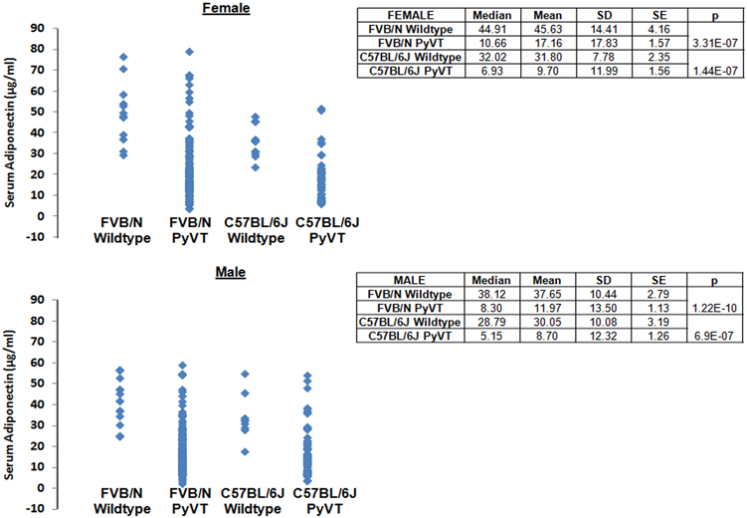
Serum adiponectin distributions in wildtype and PyVT mice. The serum adiponectin concentrations were measured by an in-house sandwich ELISA assay using blood samples collected from the tail vein of FVB/N and C57BL/6J mice. The median and mean values were calculated and displayed in the table.

**Figure 2:**
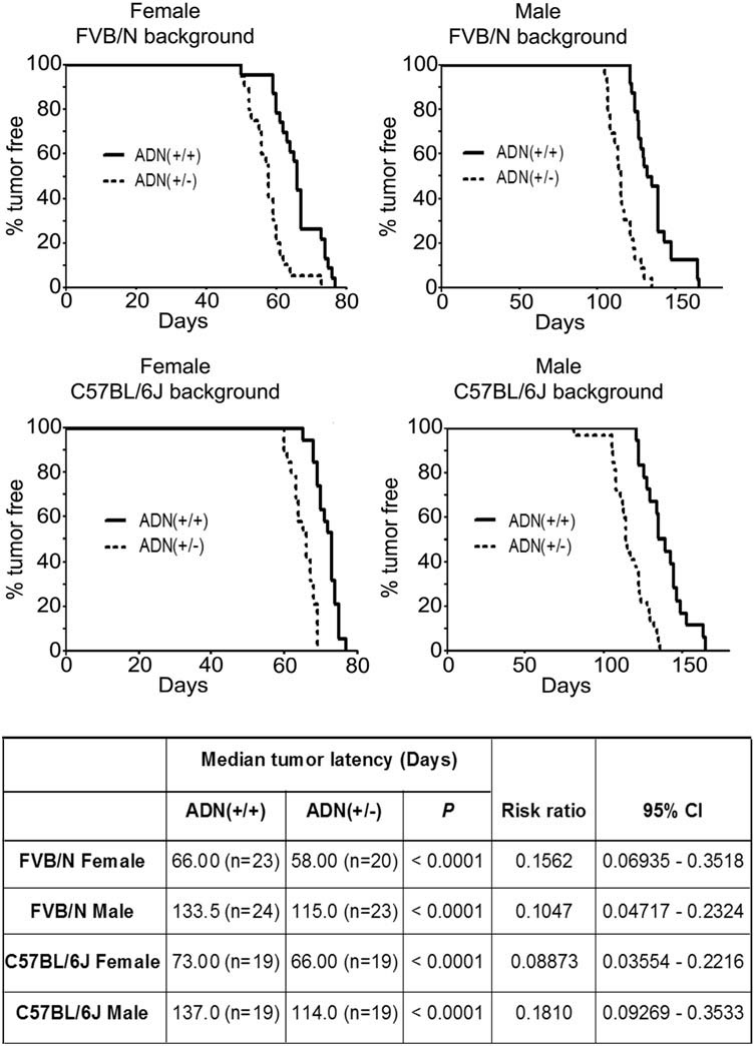
Reduced tumor latency in adiponectin haplodeficient MMTV-PyVT mice of both FVB/N and C57BL/6J genetic backgrounds. The tumor onset was closely monitored by visual inspection and palpation every 2–3 days. Latency of mammary tumors was defined as the age when a palpable lump was first detected in the mammary gland. Kaplan-Meier estimates of the tumor-free survival curves were calculated and plotted. Median value represents the time point when 50% of animals developed palpable tumor masses. The significance of differences in latency was analyzed by the Log-rank test. The comparisons were performed between *ADN(+/+)* and *ADN(+/−)* female (left panel) and male (right panel) animals in FVB/N and C57BL/6J genetic backgrounds. CI, confidence interval.

**Figure 3:**
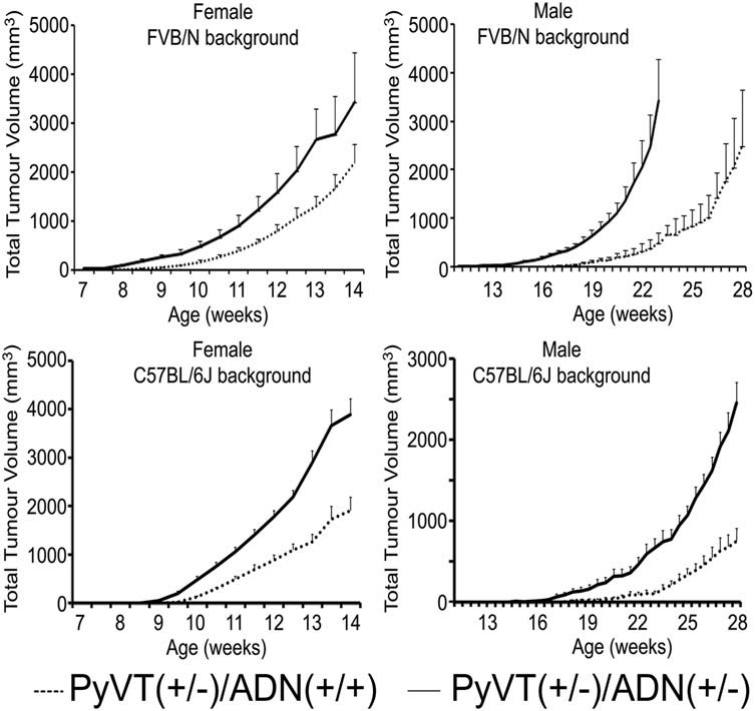
Accelerated mammary tumor development in adiponectin haplodeficient MMTV-PyVT mice. Tumor growth in *PyVT(+/−)/ADN(+/+)* and *PyVT(+/−)/ADN(+/−)* mice were monitored starting from 6 and 11 wks, up to 14 and 28 wks for female (left panel) and male (right panel) mice respectively. Tumor sizes were measured using vernier calipers and tumor volume calculated as described in Methods. Each group contained 13–20 mice, and the mean tumor volume ±SD was presented.

We generated MMTV-PyVT transgenic mice with reduced adiponectin expressions in both FVB/N and C57BL/6J backgrounds. PyVT transgenic mice with complete loss of the adiponectin alleles could not be born alive across all generations due to embryonic lethality. On the other hand, the knockout genotypes were found in male and female *PyVT(−/−)* litters. Therefore, mice with normal *PyVT(+/−)/ADN(+/+)* and reduced *PyVT(+/−)/ADN(+/−)* adiponectin expressions were used in the present study. The heterozygotes showed a 4–5 folds reduction of adiponectin levels (Figure 1), which were more relevant to those breast cancer patients with decreased adiponectin levels. Tumor development of these mice was closely monitored every 2–3 days. All mice carrying the PyVT transgene developed mammary tumors. Tumor onset was recorded as the age of the animal at which palpable abnormal masses were detected (Figure 2). The overall median age of tumor latency in *PyVT(+/−)/ADN(+/−)* mice of FVB/N background were 58 days for female (n = 20) and 115 days for male (n = 23) mice respectively, which were significantly earlier than those of *PyVT(+/−)/ADN(+/+)* mice (66 days for female and 133.5 days for male mice, n = 23 and 24 respectively, p<0.0001). Similar phenomena were also observed in mice of C57BL/6J background. The overall median tumor latency of female and male adiponectin haplodeficient PyVT mice (66 and 114 days respectively, n = 19) was significantly reduced comparing with those of mice having normal adiponectin expression levels (73 and 137 days respectively, n = 19, p<0.0001). Tumor development was monitored twice per week up to 14 and 28 weeks for female and male mice respectively (Figure 3). No tumors were found in *PyVT(−/−)* mice up to 60 weeks, irrespective of their adiponectin levels. Tumor growth was significantly accelerated in both female and male adiponectin haplodeficient PyVT mice compared to *PyVT(+/−)/ADN(+/+)* mice. At the time of sacrifice, the total wet weights of tumors in *PyVT(+/−)/ADN(+/−)* mice was over 2-fold heavier than those with normal adiponectin expression levels (Table 1). The mean tumor weight of female PyVT mice of FVB/N background when sacrificed at 14 wks of age was 9.889±3.189 g in *ADN(+/−)* animals compared to 4.483±1.645 g in *ADN(+/+)* animals. Similarly, in male FVB/N PyVT mice sacrificed at 22 wks of age, the mean tumor weight was 6.857±1.262 g in *ADN(+/−)* animals compared to that of 3.687±1.483 g in *ADN(+/+)* animals. On the other hand, although the wet weights of lung tissues in female and male *PyVT(+/−)* mice were heavier than those in non-transgenic *PyVT(−/−)* mice (data not shown), they were not significantly different between mice with reduced and normal adiponectin expressions.

**Table 1:** Total wet weights of tumor and lung tissues collected from *PyVT(+/−)/ADN(+/+)* and *PyVT(+/−)/ADN(+/−)* mice in FVB/N and C57BL/6J background.

	FVB/N				C57BL/6J			
	Female (14 weeks)		Male (22 Weeks)		Female (16 weeks)		Male (26 Weeks)	
	*ADN(+/+)* (n = 20)	*ADN(+/−)* (n = 20)	*ADN(+/+)* (n = 13)	*ADN(+/−)* (n = 18)	*ADN(+/+)* (n = 13)	*ADN(+/−)* (n = 19)	*ADN(+/+)* (n = 18)	*ADN(+/−)* (n = 18)
Tumor	4.483±1.645	9.889±3.189*	3.687±1.483	6.857±1.262*	3.609±1.846	8.704±2.742*	2.134±1.253	5.079±2.51*
Lung	0.203±0.041	0.213±0.0309	0.252±0.005	0.286±0.028	0.225±0.085	0.219±0.026	0.266±0.018	0.292±0.029

* p<0.05 vs the corresponding *PyVT(+/−)/ADN(+/+)* mice group.

### Distinct basal-like subtype of tumors in adiponectin haplodeficient PyVT mice

**Figure 4:**
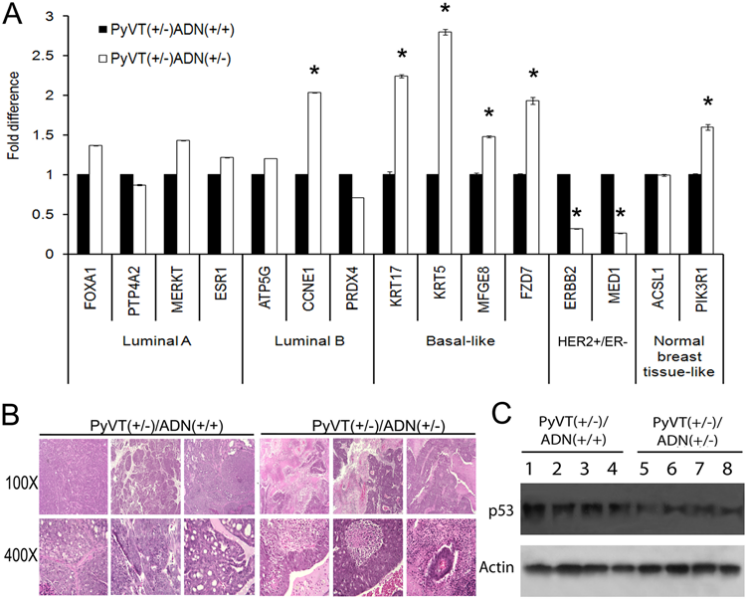
A basal-like subtype of mammary tumors derived from adiponectin haplodeficient MMTV-PyVT mice. *A*, Quantitative RT-PCR analysis of the expression levels of gene markers associated with different subtypes of breast tumors. The RNA was extracted from cultured primary tumor cells isolated from 14-week old *PyVT(+/−)/ADN(+/+)* and *PyVT(+/−)/ADN(+/−)* female FVB/N mice and quantitative PCR analysis performed as described in the Methods. *, P<0.01 vs *PyVT(+/−)/ADN(+/+)* group, n = 10. *B*, Morphological features of tumors derived from 14-week old female *PyVT(+/−)/ADN(+/+)* and *PyVT(+/−)/ADN(+/−)* mice of the FVB/N background. Distinct morphologies were observed between tumors collected from mice with normal and reduced adiponectin levels. Note that central necrosis and geographic tumor necrosis, as well as stromal lymphocytic responses represented the typical basal-like subtype of breast tumors in *PyVT(+/−)/ADN(+/−)* mice. *C*, The protein levels of p53 were much higher in tumors derived from *PyVT(+/−)/ADN(+/+)* mice compared to those of the *PyVT(+/−)/ADN(+/−)* mice as measured by Western Blotting using specific antibodies purchased from Cell Signaling Biotechnology.

Five subtypes of breast carcinoma with different outcomes, including luminal A, luminal B, HER2+/ER−, basal-like and normal breast-like, were revealed by microarray studies [42]. Luminal A and B are ER positive tumors, whereas the other three subtypes are ER negative. Our preliminary microarray analysis suggested that the molecular profiles of tumor cells derived from *PyVT(+/−)/ADN(+/−)* mice were very different from those of *PyVT(+/−)/ADN(+/+)* mice and could be clustered separately (data not shown). To further validate such an observation, gene markers associated with different tumor subtypes were quantified by real-time PCR analysis. In *PyVT(+/−)/ADN(+/−)* tumors, basal-like subtype genes, including *KRT17*, *KRT5*, *MFGE8* and *FZD7*, were significantly up-regulated, whereas HER2+/ER− subtype-related genes, *ERBB2* and *MED1*, were dramatically down-regulated (Figure 4A). Histological analysis demonstrated typical morphologic features associated with the basal-like subtype, including markedly elevated geographic tumor necrosis, ribbon-like architecture associated with central necrosis, pushing margin of invasion, and stromal lymphocytic response in tumors from *PyVT(+/−)/ADN(+/−)* mice [43] (Figure 4B). We could not detect these morphological features in any of *PyVT(+/−)/ADN(+/+)* mice or the original PyVT mice, which in contrast showed a well-structured and organized morphology, suggesting that the phenotype differences may not be tumor developmental stage dependent. Moreover, the protein levels of p53, a characteristic associated with tumors overexpressing ERBB2, was significantly higher in the *PyVT(+/−)/ADN(+/+)* tumors comparing with *PyVT(+/−)/ADN(+/−)* tumors (Figure 4C). These and the above evidence suggested that adiponectin deficiency might result in the development of a basal-like subtype tumor, which could be aroused from a different origin or subgroups of stem cells that developed tumor more aggressively.

### Accelerated growth of primary tumor cells derived from adiponectin haplodeficient mice

**Figure 5:**
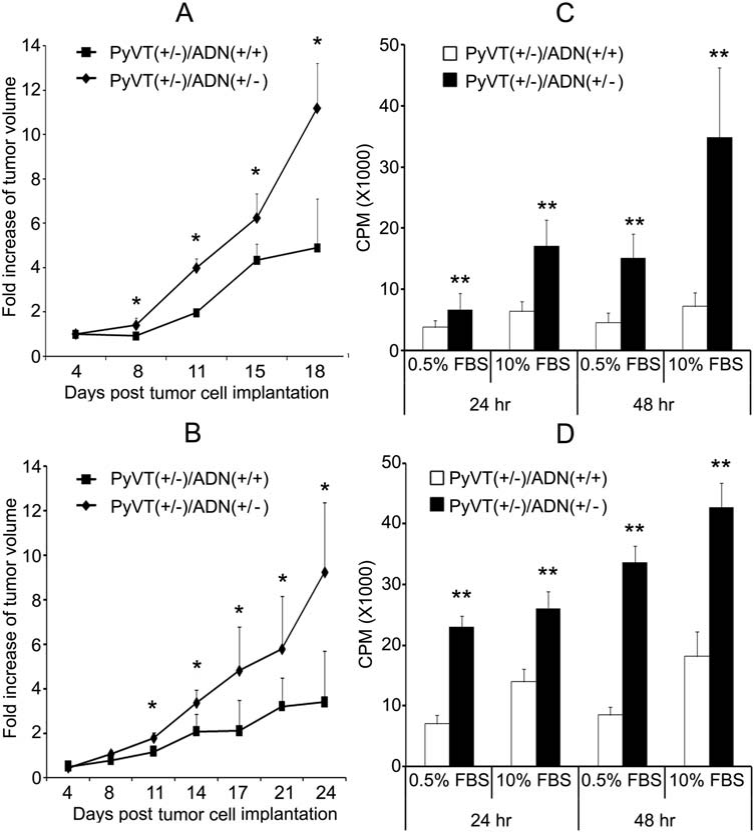
Mammary tumor cells derived from adiponectin haplodeficient mice were more aggressive. Primary mammary tumor cells were isolated from FVB/N PyVT mice with normal [*PyVT(+/−)/ADN(+/+)*] or reduced [*PyVT(+/−)/ADN(+/−)*] adiponectin expressions, and implanted into nude mice for assessing their tumor development *in vivo* (A and B), or subjected to culture and [^3^H]-thymidine incorporation assays for evaluating their proliferations *in vitro* (C and D). The comparison between *PyVT(+/−)/ADN(+/+)* and *PyVT(+/−)/ADN(+/−)* groups were performed for tumor cells derived from both female (A and C) and male (B and D) mice. Tumor *g*rowth was presented as the fold changes of tumor volume against the first measurement at day 4 (A and B). DNA synthesis was monitored in 0.5% and 10% FBS culture conditions at 24 and 48 hrs after seeding (C and D). CPM, counts per minute. *, P<0.05 and **, P<0.01 vs corresponding groups (n = 13–18).

**Figure 6:**
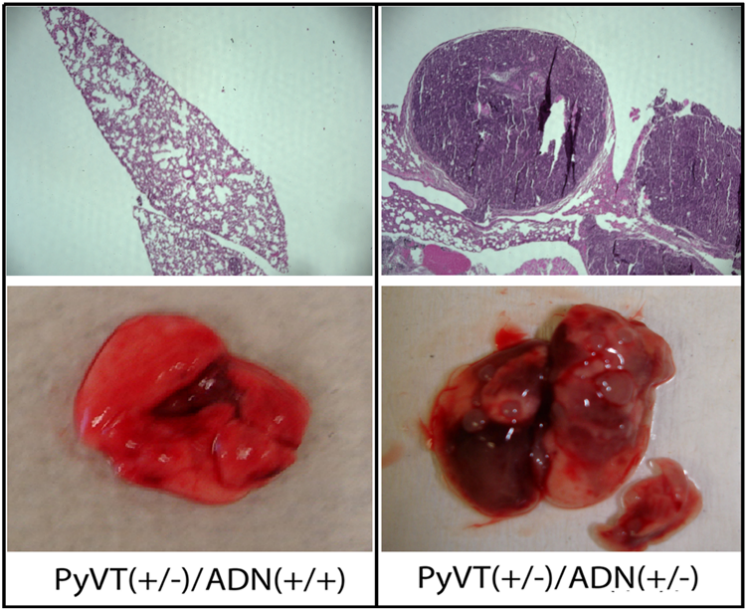
Tumor cells derived from male *PyVT(+/−)/ADN(+/−)* mice show increased metastatic capacities in nude mice comparing with those of *PyVT(+/−)/ADN(+/+)* mice. Both hematoxylin and eosin staining (upper panel) and the morphological evaluations (bottom panel) were performed to evaluate metastasis of the lung tissues.

We next isolated the primary tumor cells from the PyVT mice, and examined their tumor development in athymic nude mice following the protocol described previously [39]. Since tumors originated from different torso regions of PyVT mice might show various degrees of aggressiveness and onset latencies, we collected only tumors from the axillary mammary glands and re-implanted the isolated tumor cells into the posterior glands of athymic nude mouse by intraductal inoculation. The transplantation was reproducible and tumor growth rate correlated with the number of transplanted cells. Consistent with those observed in PyVT mice, the tumor development of cells derived from adiponectin haplodeficient mice was more aggressive than those from mice with normal adiponectin expressions (Figure 5, A and B). The accelerated tumor growth was reproducibly observed even when the cells were re-implanted for multiple times in new batches of nude mice (data not shown), suggesting that serial transplantation preserved the molecular characteristics of the tumor origin. At the time of sacrifice, the total weights of the collected tumors from *PyVT(+/−)/ADN(+/−)* mice were heavier than those of *PyVT(+/−)/ADN(+/+)* mice (Table 2). Note that when the tumor cells were exposed to physiological adiponectin secreted from adipocytes in the mammary tissue of nude mice, the change in the tumor volume was smaller, but still significantly different between *ADN(+/−)* with *ADN(+/+)* groups. At ∼3 weeks after tumor occurrence, the measurable tumor volumes were 5.5 and 2.8 fold higher in FVB/N male and female *PyVT(+/−)/ADN(+/−)* animals respectively than those of *PyVT(+/−)/ADN(+/+)* mice (Figure 3), whereas the differences for the implanted nude mice were approximately 3.2 and 2.1 fold for male and female tumor cells respectively (Figure 5). The results further suggest that the magnitude of tumor growth could be suppressed in the presence of endogenous expression of adiponectin by adipocytes. The lung tissues of mice implanted with male tumor cells showed elevated wet weights than those implanted with female tumor cells. Moreover, there was a significant difference between the two nude mice groups implanted with male *PyVT(+/−)/ADN(+/+)* and *PyVT(+/−)/ADN(+/−)* tumor cells, with much higher lung weights in the later group (Table 2). Massive lumps of metastatic tumor mass could be seen on the surface of the lungs from nude mice implanted with male *PyVT(+/−)/ADN(+/−)* tumor cells. Hematoxylin and eosin staining confirmed that the metastatic capacities of these tumor cells were much higher than those from other groups (Figure 6).

**Table 2:** Total net weights (g) of tumor and lung tissues collected from nude mice implanted with primary tumor cells isolated from female and male *PyVT(+/−)/ADN(+/+)* and *PyVT(+/−)/ADN(+/−)* mice.

	Nude mice			
	Female (18 days after implantation)		Male (24 days after implantation)	
	*ADN (+/+)* (n = 17)	*ADN (+/−)* (n = 13)	*ADN (+/+)* (n = 13)	*ADN (+/−)* (n = 18)
Tumor	0.503±0.262	0.875±0.693*	0.449±0.366	0.831±0.425*
Lungs	0.160±0.063	0.174±0.014	0.187±0.0407	0.248±0.0928*

* p<0.05 vs the corresponding *PyVT(+/−)/ADN(+/+)* mice group.

We next compared the proliferation of the isolated primary tumor cells in culture by using [^3^H]-thymidine incorporation assay (Figure 5, C and D). Cells derived from *PyVT(+/−)/ADN(+/−)* mice showed dramatically enhanced DNA synthesis under both 0.5% FBS and 10% FBS DMEM culture conditions. Moreover, the fold changes of [^3^H]-thymidine incorporation between the two time points (24 hr and 48 hr) in *ADN(+/−)* group were greater than those of *ADN(+/+)* group. Similar results were also obtained by crystal violet staining and cell number counting (data not shown). These data demonstrated that tumor cells derived from adiponectin haplodeficient mice were more aggressive, and their intrinsic properties were well preserved even under conditions without any hormonal interference.

### Elevated PI3K/Akt/beta-catenin signalling in tumor cells derived from adiponectin haplodeficient mice

**Figure 7:**
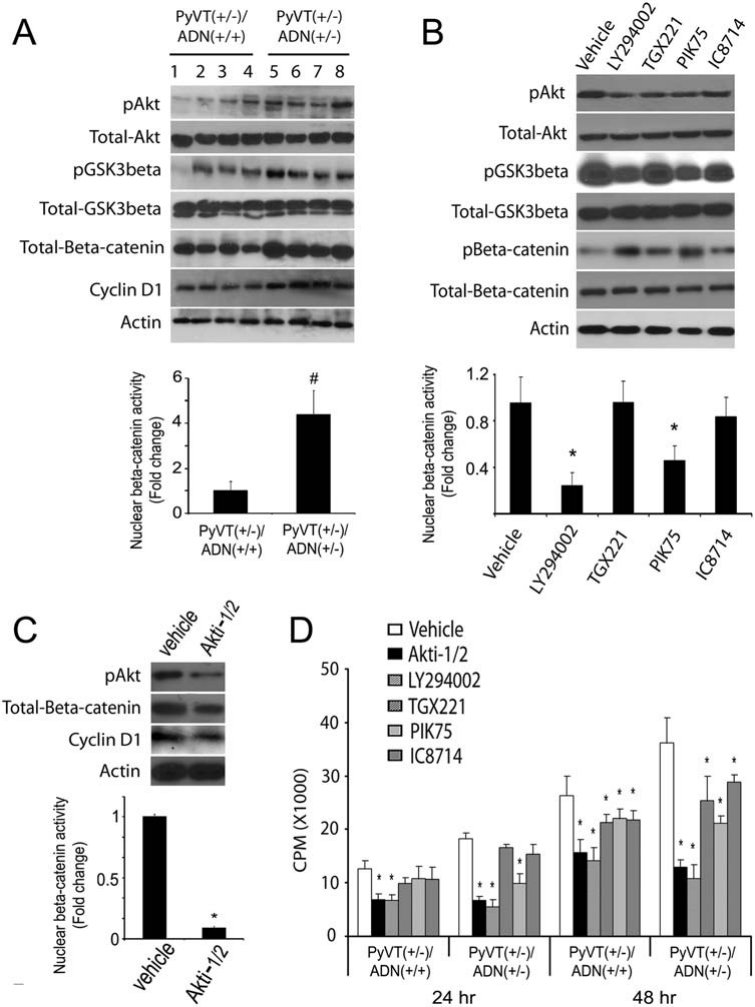
Hyperactivation of Akt/GSK3beta/beta-catenin signaling in adiponectin haplodeficient tumors. *A*, Components of the PI3K/Akt/beta-catenin axis were characterized in the tumor cell lysates by Western blotting (upper panel) and nuclear beta-catenin activities analyzed using a TOPflash/FOPflash luciferase reporter assay (bottom panel). Results were expressed as fold changes relative to the values of samples derived from *PyVT(+/−)/ADN(+/+)* cells. #, P<0.01 vs *PyVT(+/−)/ADN(+/+)* group (n = 6). *B*, Various pharmacological inhibitors, including LY294002 for PI3K, PIK-75 for p110alpha, TGX221 for p110beta and IC8714 for p110delta, were used for the treatment of *PyVT(+/−)/ADN(+/−)* tumor cells at the concentration of 10^−6^ M. The phosphorylations of Akt (pAkt), GSK3beta (pGSK3beta), and beta-catenin (pBeta-catenin), as well as their total levels in the cell samples treated with each specific inhibitor for 30 min were analyzed by Western Blotting (upper panel). After 24 hr incubation, the nuclear beta-catenin activities were evaluated using the TOPflash/FOPflash reporter assay (bottom panel). *, P<0.01 vs vehicle (n = 4). *C*, Primary tumor cells isolated from *PyVT(+/−)/ADN(+/−)* mice were cultured and treated without (vehicle) or with 10^−6^ M of specific inhibitor of Akt-1/Akt-2 isoforms (Akti-1/2) for 24 hr. Protein levels of phosphorylated Akt (pAkt), beta-catenin, and cyclinD1 in the cell lysates were analyzed by Western Blotting (upper panel) and the nuclear beta-catenin activities measured using a TOPflash/FOPflash luciferase reporter system (bottom panel). *, P<0.01 vs vehicle control (n = 3). *D*, Evaluation of the effects of various inhibitors on cell proliferation by [^3^H]-thymidine incorporation assay. CPM, counts per minute. *, P<0.01 vs vehicle in each treatment group (n = 5). Results were derived from three independent experiments.

**Figure 8:**
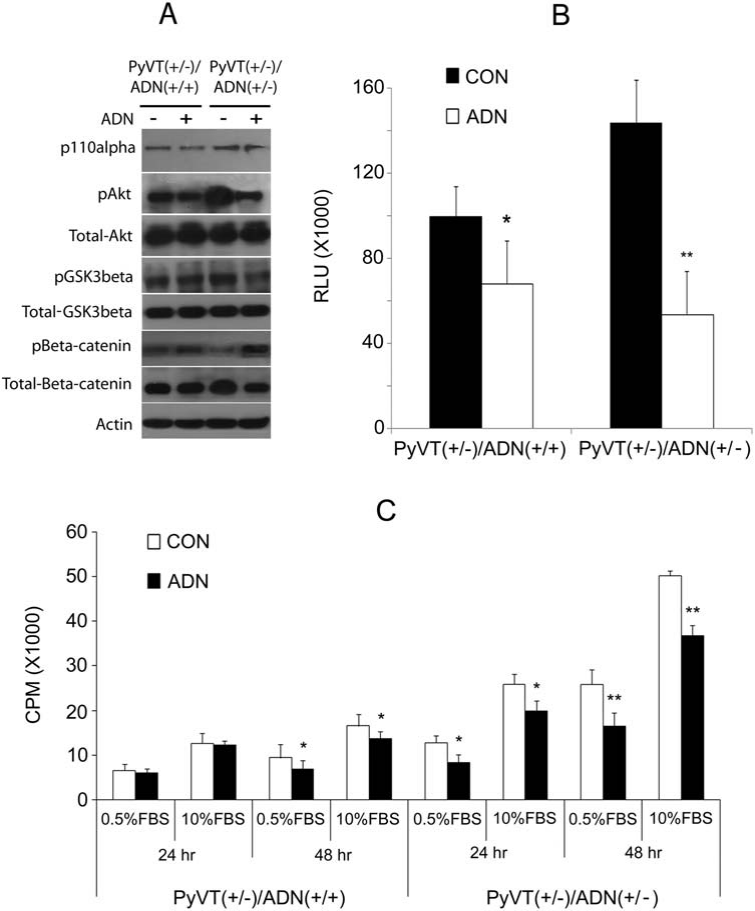
Tumor cells derived from *PyVT(+/−)/ADN(+/−)* mice showed increased sensitivity to adiponectin-mediated inhibition of Akt/GSK3beta/beta-catenin signaling and cell proliferation. Tumor cells isolated from both *PyVT(+/−)/ADN(+/+)* and *PyVT(+/−)/ADN(+/−)* mice were pre-treated with 15 µg/ml of adiponectin for 24 hr in 0.5% FBS DMEM. The serum-stimulated phosphorylation changes of Akt, GSK3beta and beta-catenin were analyzed by Western Blotting (A) as described previously [28]. The nuclear beta-catenin activities were assayed using the TOPflash/FOPflash reporter assay (B). Cell proliferation was evaluated for both types of tumor cells under the indicated treatment conditions using ^3^H-thymidine incorporation assay (C). *, P<0.05 and **, P<0.01 vs corresponding vehicle control (n = 3, from three independent experiments).

We previously reported that chronic treatment of adiponectin could modulate GSK3beta/beta-catenin pathway in MDA-MB-231 human breast cancer cells [28]. To investigate whether adiponectin inadequacy could enhance beta-catenin signaling in mammary tumors, we examined the phosphorylation status of GSK3beta and its upstream protein kinase Akt, as well as the protein levels and nuclear activities of beta-catenin (Figure 7A). The results revealed that in primary tumor cells derived from *PyVT(+/−)/ADN(+/−)* mice, phosphorylations of both Akt at serine 473 and GSK3beta at serine 9 were significantly increased. On the other hand, the phosphorylation of ERK1/2 was not different between the two types of tumor cells from *PyVT(+/−)/ADN(+/+)* and *PyVT(+/−)/ADN(+/−)* mice (data not shown). The protein levels of beta-catenin and its target cyclin D1 were largely elevated. The augmented beta-catenin signaling was also confirmed by measuring its nuclear activities, which were increased by ∼4.5 folds in *PyVT(+/−)/AND(+/−)* tumor cells according to the results from the TOPflash/FOPflash reporter assays (Figure 7A). Inappropriate Akt activation can occur through PI3K. We found that the protein levels of the p110alpha subunits of PI3K, the main isoform involved in oncogenesis, were slightly increased in *PyVT(+/−)/ADN(+/−)* tumor cells (data not shown). Both general (LY294002) and selective pharmacological antagonists against different isoforms of p110 catalytic subunits (p110alpha-selective inhibitor PIK75, p110beta-selective inhibitor TGX221 and p110delta-selective inhibitor IC8714) [34] were then used for testing their effects in cells isolated from *PyVT(+/−)/ADN(+/−)* tumors. Treatment with either LY294002 or PIK75 led to significantly attenuated phosphorylations of Akt and GSK3beta and more than 50% reductions of nuclear beta-catenin activities, whereas treatment with IC8714 and TGX221 did not have much impacts (Figure 7B). Similarly, treatment with a specific inhibitor of Akt1 and Akt2 (Akti-1/2) significantly reduced beta-catenin and cyclin-D1 expression levels and caused about 11-fold decrease of nuclear beta-catenin activities (Figure 7C). To further verify the involvement of PI3K and Akt in the accelerated proliferation of tumor cells derived from *PyVT (+/−)/ADN(+/−)* mice, their inhibitors were used for cell proliferation measurement using [^3^H]-thymidine incorporation assay. Importantly, the general inhibitor LY294002 and Akti-1/2 showed greater extent of attenuation on the cell growth at all time points, whereas the p110alpha-selective inhibitor PIK75 was more potent than the other two inhibitors (Figure 7D), suggesting that blockade of PI3K or Akt reversed the proliferative advantage of adiponectin haplodeficient tumors. Adiponectin treatment significantly attenuated phosphorylations of Akt and GSK3beta and beta-catenin protein levels and nuclear activities, as well as inhibited cell proliferation to a greater extent in *PyVT (+/−)/ADN(+/−)* tumor cells (Figure 8). On the other hand, it had little effects on p110alpha levels. These results implicated that the activation of PI3K/Akt pathway might contribute to the elevated beta-catenin signalling cascades in adiponectin haplodeficient mammary tumors.

### Decreased PTEN activities caused by altered redox environment in adiponectin haplodeficient PyVT tumors

**Figure 9:**
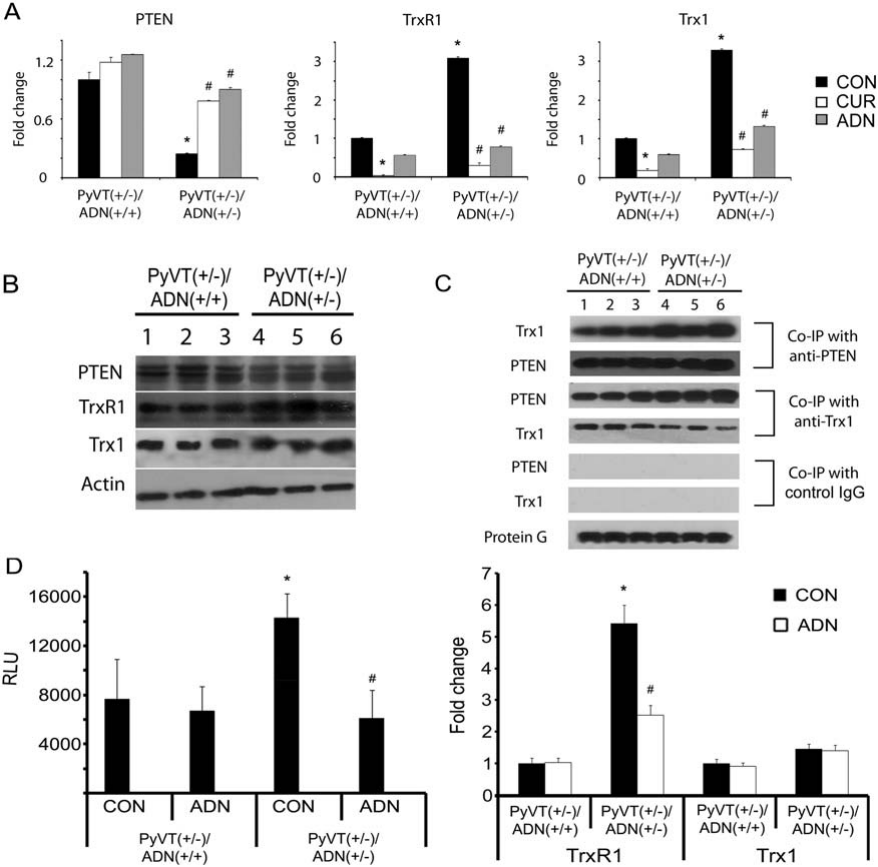
Inactivation of PTEN was at least partially caused by the augmented Trx1/TrxR1 redox activities in *PyVT(+/−)/ADN(+/−)* tumor cells. *A*, activities of PTEN, TrxR1 and Trx were evaluated in the lysates derived from *PyVT(+/−)/ADN(+/+)* and *PyVT(+/−)/ADN(+/−)* tumor cells using colorimetric assays. Briefly, cells were treated with vehicle control (CON), 10 µM curcumin (CUR) or 15 µg/ml adiponectin (ADN) for 24 hrs. Immunoprecipitation and phosphatase assay were performed as described in Methods. Results were expressed as fold changes relative to the enzyme activities in *PyVT(+/−)/ADN(+/+)* tumor cells treated with vehicle control. *B*, total protein levels of PTEN, TrxR1 and Trx1 in cell lysates from *PyVT(+/−)/ADN(+/+)* and *PyVT(+/−)/ADN(+/−)* tumors were analyzed by Western Blotting. *C*, co-immunoprecipitations were performed with the specific antibodies against PTEN or Trx1 in both *PyVT(+/−)/ADN(+/+)* and *PyVT(+/−)/ADN(+/−)* tumor cell lysates. The immune-complexes were analyzed by SDS-PAGE and Western Blotting using antibodies as indicated. *D*, intrinsic transcriptional activities of TrxR1 promoter (left panel) and the mRNA levels of TrxR1 and Trx1 (right panel) were evaluated in *PyVT(+/−)/ADN(+/+)* and *PyVT(+/−)/ADN(+/−)* tumor cells treated without (CON) or with adiponectin (ADN, 15 µg/ml) using TrxR1 reporter assay (left panel) and quantitative RT-PCR respectively (right panel). *, P<0.05 vs *PyVT(+/−)/ADN(+/+)* cell control; #, P<0.05 vs *PyVT(+/−)/ADN(+/−)* cell control (n = 3, from three independent experiments).

PTEN is one of the most frequently mutated tumor suppressors that can prevent the activation of the cell survival PI3K/Akt signaling pathway [44]. In the absence of PTEN function, cells exhibit elevated Akt activities. It has been reported that PTEN could bind to Trx1 in the cytosol, resulting in a functional loss of its lipid phosphatase and membrane binding activity [45]. Interestingly, PTEN activities were decreased by more than 50% in *PyVT (+/−)/ADN(+/−)* tumor cells (Figure 9A), whereas its total protein amount was not significantly different (Figure 9B). The activities of both Trx1 and its upstream binding enzyme, TrxR1, were augmented by nearly 40% in *PyVT(+/−)/ADN(+/−)* tumor cells (Figure 9A). While the protein levels of Trx1 were similar between *PyVT(+/−)/ADN(+/+)* and *PyVT(+/−)/ADN(+/−)* tumors, the total amount of TrxR1 was increased in *PyVT(+/−)/ADN(+/−)* tumor cells (Figure 8B). Surprisingly, co-immunoprecipitation experiment revealed that the amounts of Trx1-bound PTEN were dramatically increased in tumor cells derived from the adiponectin haplodeficient *PyVT(+/−)* mice (Figure 9C). Treatment with curcumin, an irreversible inhibitor of TrxR1 (40), elevated PTEN activity by nearly 3 folds in *PyVT(+/−)/ADN(+/−)* tumor cells, which was accompanied by the decreased activities of both TrxR1 and Trx1 (Figure 9A). A stimulatory effect on PTEN activity was also observed in cells treated with adiponectin (Figure 9A). In *PyVT(+/−)/ADN(+/−)* tumor cells, the *TrxR1* promoter-driven reporter activity was ∼1.8 fold higher than that of *PyVT(+/−)/ADN(+/+)* tumor cells (Figure 9D). Treatment with adiponectin for 24 hrs significantly reduced the reporter activities by ∼60% in *PyVT(+/−)/ADN(+/−)* tumor cells but had no significant effects on *PyVT(+/−)/ADN(+/+)* tumor cells. Similar effects were also observed for *TrxR1* mRNA levels in tumor cells treated with or without adiponectin (Figure 9D). Taken together, these results suggested that in tumor cells derived from adiponectin haplodeficient mice, the increased TrxR1/Trx1 redox activities might be involved in inactivation of PTEN and hyperactivation of PI3K/Akt signalling pathways.

## Discussion

Although the anti-tumor activities of adiponectin have been suggested by numerous clinical and experimental evidences, the underlying mechanisms remain to be established. Here, we have evaluated the impacts of reduced adiponectin expression on mammary tumor development in MMTV-PyVT mice. Our results demonstrate that in both FVB/N and C57BL/6J genetic backgrounds, adiponectin inadequacy is associated with earlier tumor onset and accelerated tumor growth (Figure 2, Figure 3 and Table 1). The aggressive phenotypes of tumor cells derived from *PyVT(+/−)/ADN(+/−)* mice are retained during serial trans-implantations in nude mice as well as multiple passages in culture (Figure 5), suggesting that insufficient adiponectin production from adipose tissue might result in an abnormal microenvironment that leads to distinct but permanent genetic and phenotypic modifications of mammary epithelial cells. Indeed, both histological and gene expression analyses suggest a distinct basal-like subtype of tumors in *PyVT(+/−)/ADN(+/−)* mice (Figure 4). Tumor cells derived from adiponectin haplodeficient mice show enhanced beta-catenin nuclear activities and protein stabilities, which might be partly attributed to the hyperactivated PI3K/Akt signaling (Figure 7). While adiponectin treatment restores most of the changes downstream of PI3K, including Akt and GSK3beta and beta-catenin, it has little effects on p110alpha (Figure 8). On the other hand, adiponectin deficiency causes inactivation of PTEN and results in the hyper-activated PI3K/Akt signaling in *PyVT(+/−)/ADN(+/−)* tumors (Figure 9).

PTEN/PI3K/Akt constitutes an important pathway regulating the signaling of multiple biological processes such as apoptosis, metabolism and cell proliferation [46]. PTEN activity is lost by mutations, deletions, promoter methylation silencing, or protein modifications at high frequency in many primary and metastatic human cancers [47]. Hyperactivation of the PI3K/Akt signaling pathway triggered by PTEN inactivation has been found to correlate with increased breast cancer risks, poor prognosis and resistance to hormone therapy [44]. In adiponectin haplodeficient PyVT tumors, while the total protein levels of PTEN are not altered, its activities are significantly decreased (Figure 9). PTEN is well-known to be regulated by the redox state of the active site cysteine residues [48]. Oxidation of PTEN resulted from thiol modification leads to reversible inhibition of its phosphatase activity. The thioredoxin system, composed of TrxR, Trx, and NADPH, represents one of the main thiol-dependent electron donor systems and plays critical roles in the regulation of the cellular redox environment [49]. Although the reduction of oxidized PTEN appears to be dominantly mediated by Trx, it has been reported that Trx1 inhibits its phosphatase activity by binding in a redox dependent manner to PTEN through disulfide bond formation [45]. Moreover, knocking out of thioredoxin-interacting protein, an inhibitor of Trx NADPH-dependent reduction of PTEN, causes accumulation of oxidized PTEN and elevated Akt phosphorylation [50]. We find that there is a significantly augmented formation of Trx1-PTEN complexes in tumor cells derived from adiponectin haplodeficient PyVT mice, possibly due to elevated TrxR1 and Trx1 activities (Figure 9A). Adiponectin treatment decreases TrxR1 promoter-mediated transcription and its mRNA levels, which are highly upregulated in adiponectin haplodeficient tumors (Figure 9D). These results suggest that adiponectin might regulate PTEN activities through Trx/TrxR redox system, and an altered Trx/TrxR redox balance could play important roles in promoting tumorigenesis in *PyVT(+/−)/ADN(+/−)* mice.

In this study, we have shown that inhibition of both PI3K and Akt results in a reduced nuclear beta-catenin activities and protein stabilities, supporting the involvement of PI3K/Akt pathway in the hyper-activation of beta-catenin signalling associated with adiponectin haplodeficient tumors (Figure 7). Notably, the cross-talks between PI3K/Akt and the canonical Wnt/beta-catenin signaling pathways have been demonstrated by a number of studies from independent groups (23). Overexpression of PTEN inhibits Wnt-1 induced beta-catenin stabilization and mammary tumorigenesis in mice [51]. PI3K/Akt pathway is involved in Wnt3a-induced proliferation and beta-catenin nuclear accumulation in NIH3T3 cells [52]. In HT29 colorectal adenocarcinoma cells, inhibition of PI3K was accompanied by a considerably reduced expression level of beta-catenin [53]. The linkage between Wnts and PI3K/Akt signalling have also been found in the regulation of bone mass, osteoblast progenitor proliferation, differentiation and osteoblast apoptosis, as well as cardiomyogenesis [54]. The protein levels of p110alpha subunits are elevated in tumor cells isolated from adiponectin haplodeficient PyVT mice. However, adiponectin treatment has no effects on p110alpha, despite that it can inhibit Akt phosphorylation and nuclear beta-catenin activities (Figure 7), suggesting that the inhibitory effects of this hormone is downstream of PI3K and upstream of Akt, possibly through modulating PTEN's activities.

Insufficiency in adiponectin production might promote mammary tumor formation from distinct type of cells, as suggested by the consistent morphological and gene expression differences between tumors derived from *PyVT(+/−)/ADN(+/+)* and *PyVT(+/−)/ADN(+/−)* mice (Figure 3). Adiponectin haplodeficient tumor is more related to a basal-like subtype, which is characterized by high proliferative activity and unfavorable prognosis. The origin of this subtype tumor is unclear, but suggested to be the basal/myoepithelial cells, derived from epithelial-to-mesenchymal transition as a result of dedifferentiation, or from stem cells [43]. It will be interesting to investigate which types of tumor cell transformation could be facilitated by the altered microenvironment associated with adiponectin haploinsufficiency. It has long been noticed that cancer cells exhibit increased glycolysis for ATP production due, in part, to respiration injury (the Warburg effect). The increase in NADH caused by respiratory deficiency inactivates PTEN through a redox modification mechanism, leading to Akt activation. Our group has recently reported that adiponectin deficiency leads to dysregulated mitochondrial functions, which result in decreased activities of the respiratory chain and subsequent accumulation of reactive oxygen species [55]. We have also found that adiponectin can modulate redox-regulated transcription factor Sp1 activities [56]. Interestingly, the expression of both Trx1 and TrxR1 can be regulated by Sp1 [57]. Whether these mechanisms contribute to the dysregulated Trx/TrxR redox system in adiponectin insufficiency-related carcinogenesis are currently under investigation in our laboratory. Nevertheless, these findings might provide a novel mechanistic insight to explain how metabolic alteration in adiponectin haplodeficient tumor may gain a survival advantage.

## Materials and Methods

### Materials

Antibodies against PI3K p110-alpha (#4255), PI3K p85 (#4292), phospho-Akt (Ser473) (#9271), Akt (#9272), GSK3beta (#9315), phospho-GSK3beta (Ser9) (#9336) and phospho-beta-catenin (Ser33/37/Thr41) (#9561) were obtained from Cell Signaling Biotechnology (Beverly, MA). Anti-Trx1 (sc-20146), anti-TrxR1 (sc-28321), and anti-beta actin (sc-1615) antibodies were from Santa Cruz Biotechnology (Santa Cruz, CA). Anti-PTEN (MAB4037) was from Chemicon International, Inc. (Temecula, CA), anti-Cyclin D1 (CC12) was from CalBiochem-Novachem Crop. (San Diego, CA), and sheep anti-beta-catenin was from Symansis (Auckland, New Zealand). Pharmacological inhibitors, including Akt-1/2 inhibitor, PI3K p110alpha inhibitor PIK-75, PI3K p110beta inhibitor TGX221, and PI3K p110delta inhibitor IC87114 were provided by Dr Peter R. Shepherd [34], [35]. The general PI3K inhibitor, LY294002, was from Cell Signaling Biotechnology. ImProm-II™ Reverse Transcription System and Bright-GloTM luciferase assay system were from Promega (Madison, WI). TOP/FOPflash (T-cell factor-lymphoid enhancer factor-1 (TCF-LEF) reporter plasmid) was from Upstate (Lake Plasid, NY). pGL-TrxR1 reporter plasmid was generated by cloning the proximal promoter of the human TrxR1 gene using the GenomeWalker kit from Clontech (Palo Alto, CA). The human TrxR1 and Trx1 were purchased from Sigma. The rat TrxR1 was purified from rat liver according to published procedure [36] and the purity confirmed by mass spectrometry analysis. Unless specified, all chemicals were obtained from Sigma-Aldrich Co. (St Louis, MO). Recombinant full length adiponectin (ADN) was produced as we described previously [28].

### Establishment of the MMTV-PyVT transgenic mice haplodeficient in adiponectin expression

FVB/N-Tg(MMTV-PyVT)634Mul/J transgenic mice were obtained from the Jackson Laboratory (Bar Harbor, Maine) [37]. Since the female PyVT transgenic mice were defective in litter delivery and lactation, all breedings were carried out using male PyVT transgenic mice. The male heterozygote *PyVT(+/−)* mice were cross-bred with female adiponectin knockout mice [38] and back-crossed for at least 12 generations to obtain mice with reduced adiponectin expression in both C57BL/6J and FVB/N backgrounds. The genotype was verified by PCR analysis of their genomic DNA using primers listed in Table 3. In addition, serum adiponectin levels were monitored using an in-house ELISA, with the standard curve generated from known concentrations of recombinant adiponectin. Note that mice with the genotype of *PyVT(+/−)/ADN(−/−)* (transgenic PyVT with adiponectin null alleles) could not be found in all generations of alive litters, which included over 800 mice. On the other hand, their embryos were found to be dead at the early stage of foetal development. As a consequence, the sizes of litters with abnormal adiponectin expressions (3–5) were consistently smaller when compared to those of control PyVT breeding pairs (8–10). Therefore, the PyVT transgenic mice with adiponectin deficiency were referred to those with *PyVT(+/−)/ADN(+/−)* genotypes in this study. The circulating levels of adiponectin in *PyVT(+/−)/ADN(+/−)* FVB/N and C57BL/6J mice range from 3–15 µg/ml and 0.2–5 µg/ml respectively, whereas *PyVT(+/−)/ADN(+/+)* mice in both FVB/N and C57BL/6J background have a much higher adiponectin level of over 20 µg/ml and 10 µg/ml respectively, with the median values increased by 4–5 folds. Tumor development was closely monitored every 2–3 days. Tumor latency was recorded as the age of mice when palpable tumors were first detected in at least one of the ten mammary fat pads. Tumor sizes were measured using digital vernier calipers and tumor volume calculated using the formula [sagittal dimension (mm)×(cross dimension (mm)^2^] / 2 and expressed in mm^3^. All animal experimental protocols were approved by the Animal Ethics Committee at the University of Hong Kong and their care was in accord with the institution guidelines.

**Table 3:** List of primers used for genotyping.

Primer name	NCBI GeneBank accession IDs	Sequence range	Product size (bp)	Primer sequences
AdipoWT	NT_039624	11673–12146	473	(F) 5′- CCA GAG AAC AAC GAA CAA GGA- 3′
				(R) 5′ – CGA ATG GGT ACA TTG GGA AC- 3′
Neo	User_PGKneobpA Sequence sequence 4575 bp DNA circular SYN 08/24/2007	2950–3101	171	(F) 5′ – TGA ATG AAC TGC AGG ACG AG- 3′
				(R) 5′ – ATA CTT TCT CGG CAG GAG CA- 3′
MMTV/PyVT	J02288	881–1437	556	(F) 5′- GGA AGC AAG TAC TTC ACA AGG G- 3′
				(R) 5′- GGA AAG TCA CTA GGA GCA GGG- 3′
Tcrd	NG_007044	1715433–1715638	206	(F) 5′- CAA ATG TTG CTT GTC TGG TG- 3′
				(R)5′ GTC AGT CGA GTG CAC AGT TT- 3′

### Sandwich ELISA for murine adiponectin

The anti-murine adiponectin monoclonal antibody was biotinylated with a kit from Pierce, and free biotin was removed by dialysis. The polyclonal anti-murine adiponectin antibody was diluted to a concentration of 2 µg/ml, added to each well of a microtiter plate, and incubated overnight at 4 °C. The coated plate was washed 3 times with PBS containing 1% bovine serum albumin and blocked with 100 µl of PBS containing 1% bovine serum albumin and 0.05% Tween for 2 h. Mouse serum was diluted 1∶10000, and 100 µl of the diluted samples were applied to each well along with the standard, incubated at 37 °C for 1 h, washed 3 times with PBS-Tween, and then incubated with 100 µl of the biotinylated monoclonal antibody (2 µg/ml) for another 2 h. After washing 3 times, the wells were incubated with streptavidin-conjugated horseradish peroxidase for 60 min and subsequently reacted with tetramethylbenzidine reagent for 15 min. 100 µl of 2 M H_2_SO_4_ was added to each well to stop the reaction, and the absorbance at 450 nm was measured. The intra- and interassay coefficients of variance were determined by measuring five plasma samples in a total of six independent assays with duplicate determinations.

### Primary tumor cell isolation, culture and re-implantation

Primary cell isolation was performed as described previously with slight modifications [39]. Briefly, aseptically collected tumors from PyVT mice were mechanically minced, passaged through a 100-µm sterile nylon cell strainer (BD Falcon) and suspended in serum-free high glucose DMEM. Cells were further dissociated by serial passaging through a syringe with 25-gauge needles. After brief centrifugation at 1,000 r.p.m for 5 minutes to remove dead cell debris and the low-density stromal cells, the cell pellets were resuspended for viable cell counting using 0.4% trypan blue. 10^6^ of isolated primary tumor cells were implanted into the third right mammary fat pad of female athymic nu/nu mice (4–6 weeks) by intraductal injection. Tumor development was monitored every 3–4 days using caliper measurements (in millimeters) in two perpendicular dimensions (length and width). Tumor volumes were calculated as described above.

### Co-immunoprecipitation and Western Blotting

Isolated tumor tissues were homogenized in RIPA buffer [50 mM Tris-HCl, pH 7.4; 1 mM EDTA; 150 mM NaCl; 1% Nonidel P40; 1% Triton X-100; 0.5% deoxycholic acid sodium salt; 1 mM NaF; 1 mM sodium orthovanadate; and complete protease inhibitor cocktail (Roche Applied Science, IN)] on ice and centrifuged for 5 min at 14,000 r.p.m to remove large debris. Protein concentration of the supernatant was determined by a BCA Protein Reagent Kit (Pierce Biotechnology, Rockford, IL). Five hundred micrograms of the total cell lysates were firstly incubated with rabbit IgG for 30 minutes, pre-cleared with 50 µl of protein G-Sepharose beads (Pierce Biotechnology, Rockford, IL), and then incubated with two micrograms of either Anti-Trx1 or Anti-PTEN antibody overnight at 4°C. 50 µl of protein G-Sepharose beads was added and incubated for 2 hrs at 4°C. Beads bound with immune complexes were collected by centrifugation and washed twice prior to elution into 90 µl of buffer containing 0.2 M Glycine-HCl, pH 2.5, which was neutralized with 10 µl of neutralization buffer (1 M Tris-HCl, pH 9.0). The elutants were subjected to 15% SDS-PAGE and Western blotting analysis, or enzyme activity measurement as described below.

For Western Blotting, fifty micrograms of proteins derived from cell or tissue lysates were separated by SDS-PAGE and transferred to polyvinylidene difluoride membranes. Following blocking, membranes were probed with various primary antibodies to determine different levels of protein expressions. Immunoreactive antibody-antigen complexes were visualized with the enhanced chemiluminescence reagents from GE Healthcare (Uppsala, Sweden).

### [^3^H]-thymidine incorporation assay for cell proliferation measurement

5×10^4^ of isolated primary tumor cells were seeded into each of the 24-well culture plate and allowed a period of at least 24 hours for cell settlement and attachment. After being treated under different conditions, 1 µCi/ml of [^3^H-methyl] thymidine was added into each well for 6 hours of incorporation. At the end of experiment, the culture media were removed and cells washed twice with cold PBS. DNA was precipitated with 0.5% trichloroacetic acid for 30 min. Air-dried precipitates were then solubilized with 0.2 mol/l NaOH, neutralized with 0.2 mol/l HCl, and incorporated [^3^H]-thymidine was quantified with a liquid scintillation counter (Backman LS6500).

### Quantitative RT-PCR

Total RNA was isolated from primary tumor cells and used for the synthesis of cDNA. Quantitative RT-PCR was performed using SYBR® GreenER™ qPCR Supermix (Invitrogen, Carlsbad, CA). The reactions were carried out on a 7000 Sequence Detection System (Applied Biosystems, Foster City, CA). Quantification was achieved using Ct values that were normalized with 18S RNA as internal control. The primers were listed in Table 4.

**Table 4:** List of primers used for real time quantitative PCR analysis.

Gene name	Gene symbol	Accession IDs	Sequence range	Product size (bp)	Primer sequences
Forkhead box A1	*FOXA1*	NM_008259	456–588	133	(F) 5′-GAA GGG CTC CTG TGC TAG TT-3′
					(R) 5′-AGG ACA TGT TGA AGG AAG CC-3′
Protein tyrosine phosphatase 4a2	*PTP4A2*	NM_008974	427–598	172	(F) 5′-GAA GGG CTC CTG TGC TAG TT-3′
					(R) 5′-TGC CCA TTG GTA TCT CTG AA-3′
c-mer proto-oncogene tyrosine kinase	*MERKT*	NM_008587	1944–2127	185	(F) 5′-AAG CAG CAT GCA TGA AAG AC-3′
					(R) 5′-TGC AGG TGA ATG TAC TTG GG-3′
Estrogen receptor 1 (alpha)	*ESR1*	NM_007956	1560–1752	193	(F) 5′-CCG GAG TGT ACA CGT TTC TG-3′
					(R) 5′-TTG TTA CTC ATG TGC CGG AT-3′
ATP synthase, H+ transporting, mitochondrial F0 complex, subunit c	*ATP5G*	NM_007506	167–223	57	(F) 5′-GGG AAT TCC AGA CCA GTG TC-3′
					(R) 5′-TTG AGA GAT GGG TTC CTG GC-3′
Cyclin E1	*CCNE1*	NM_007633	160–330	171	(F) 5′-ACA GCT TCG GGT CTG AGT TC-3′
					(R) 5′-GGC AAT TTC TTC ATC TGG GT-3′
Peroxiredoxin 4	*PREDX4*	NM_016764	716–883	118	(F) 5′-CGA TGA CAA AGG AGT CCT GA-3′
					(R) 5′-GCT GGA TCT GGG ATT ATT GT-3′
Keratin 17	*KRT17*	NM_010663	679–851	173	(F) 5′-AAG AAG AAC CAC GAG GAG GA-3′
					(R) 5′-AAG AAC CAG TCT TCG GCA TC-3′
Keratin 5	*KRT5*	NM_027011	1005–1170	166	(F) 5′-GCA GAC ACA CGT CTC TGA CA-3′
					(R) 5′-TTG CAG CTC CTC ATA CTT GG-3′
Milk fat globule-EGF factor 8 protein	*MFGE8*	NM_001045489	278–444	167	(F) 5′-AGA CTG AGA GAG GAC CAT GC-3′
					(R) 5′-CAT GCC CAG CTG TGT AGA AC-3′
Frizzled homolog 7	*FZD7*	NM_008057	1477–1631	155	(F) 5′-TTC CTA GGT GAG CGT GAC TG-3′
					(R) 5′-TAG GTG AGC ACC GTG AAG AG-3′
Chemokine (C-X-C motif) ligand 1	*CXCL1*	NM_008176	179–352	174	(F) 5′-ACC CAA ACC GAA GTC ATA GC-3′
					(R) 5′-GTT GTC AGA AGC CAG CGT T-3′
v-erb-b2 erythroblastic leukemia viral oncogene homolog 2	*ERBB2*	NM_001003817	2980–3162	183	(F) 5′-ATT TGC TGG AGA AGG GAG AA-3′
					(R) 5′-AGT CCT CGT TCT GGA TGA CC-3′
Mediator complex subunit 1	*MED1*	NM_013634	1459–1623	165	(F) 5′-CAG ACC TTG GAG TGA AAC CA-3′
					(R) 5′-GAG CCC AGT CCA TTC TGT CT-3′
Acyl-CoA synthase long-chain family member 1	*ACSL1*	NM_007981	1586–1759	174	(F) 5′-CTA TGA AGG CTA CGG ACA GA-3′
					(R) 5′-CCT TTC ACA CAC ACC TCA CC-3′
Phosphatidylinositol 3-kinase, regulatory subunit, polypeptide 1 (p85 alpha)	*PIK3R1*	NM_001077495	1906–2080	175	(F) 5′-TCC AAA TAC CAG CAG GAT CA-3′
					(R) 5′-ATG CTT CGA TAG CCG TTC TT-3′
Thioredoxin reductase 1	*TXNRD1*	NM_00142523	1587–1763	177	(F) 5′-TTG GAA TAT GGC TGT TGT GG-3′
					(R) 5′-CAC GAC ACG TTC ATC GTC TT-3′
Thioredoxin 1	*TXN1*	NM_011660	315–484	170	(F) 5′-AAG CCC TTC TTC CAT TCC CT-3′
					(R) 5′-CCT TGT TAG CAC CGG AGA AC-3′
18S RNA	*RN18S*	NR_003278	1194–1294	101	(F) 5′-TAA AGG AAT TGA CGG AAG GG-3′
					(R) 5′-CTG TCA ATC CTG TCC GTG TC-3′

### Beta-catenin/T-cell factor-lymphoid enhancer factor-1 (TCF-LEF) and thioredoxin reductase 1 (TrxR1) transcription reporter assay

Nuclear activities of endogenous beta-catenin were analyzed by the TOPflash/FOPflash reporter system as described previously [28]. To normalize transfection efficiency in the reporter assays, cells were cotransfected with pRL-TK plasmid, which contains a functional *Renilla* luciferase gene cloned downstream of a herpes simplex virus thymidine kinase promoter (Promega, Madison, WI). The luciferase reporter containing a human thioredoxin reductase promoter region (from −386 bp to +218 bp, pGL3-TR) was constructed into a firefly-luciferase pGL3-basic Vector (Promega, Madison, WI). The assay was carried out as described above with the original unmodified pGL3-basic vector as a negative control. Luminescence was measured using a Bright-Glo™ Luciferase Assay System (Promega, Madison, WI) on Lumat LB9507, and normalized to control *Renilla* luciferase signal. Luciferase activity was calculated against the negative control signals and fold differences were compared among groups in separate assays.

### Measurement of PTEN lipid phosphatase activities

The lipid phosphatase activity of PTEN was measured as described previously with slight modifications [40]. Phosphatase reactions were performed in 25 µl assay buffer (100 mM Tris-HCl pH 8, 10 mM DTT, and 200 µM water-soluble diC8-PIP3) with 25 µl sample. PTEN proteins immunoprecipitated on protein G-Sepharose beads (Pierce Biotechnology, Rockford, IL) were washed twice in a low stringency buffer (20 mM HEPES, pH 7.7, 50 mM NaCl, 0.1 mM EDTA and 2.5 mM MgCl_2_) and once in the phosphatase assay buffer lacking PIP3. Reactions were done in a 96-well plate with an incubation period of 40 minutes at 37°C. The release of phosphate from the substrate was measured in a colorimetric assay by using the Biomol Green Reagent in accordance with the instructions of the manufacturer. The absorbance at 620 nm was measured with a µQuant MQX200 microplate reader (Biotek Instruments, Inc., Highland Park, VT). A standard curve was performed in each assay, and the amount of free phosphate was calculated from the standard curve line-fit data.

### Measurement of TrxR1 and Trx1 activities

The assays for measuring the activities of TrxR and Trx were performed in 96-well plates using an insulin reduction endpoint assay as described previously [41] with slight modification. For determination of TrxR1 activity, 25 µg of the primary tumor lysates were mixed thoroughly with a 50 µl reaction buffer containing 55 mM HEPES, pH 7.6, 0.02 mM insulin, 0.4 mM NADPH, 2 mM EDTA and 2 µM human Trx1 in the µQuant MQX200 microplate reader (Biotek Instruments, Inc). Reaction solutions without human Trx1 were used as the control. After performing a 20-min incubation at 37°C, 200 µl of 1 mM DTNB in 6 M guanidine hydrochloride solution was added to stop the reaction. The free thiols of the reduced insulin were determined by DTNB reduction, and the activity of TrxR was represented as the absorbance at wavelength 412 nm, where 1 mole of NADPH reduced 1 mole of disulfide, giving rise to 2 mole of free TNB with the extinction coefficient 13.6 mM^−1^ cm^−1^. For accessing the activity of Trx, assays were performed as above with the similar reaction cocktail except that 600 nM rat TrxR1 but not Trx1 was included. Reaction solutions without TrxR1 were used as the control.

### Data analysis and statistics

All experiments were performed with six to eight samples per group, and all results were derived from at least three independent experiments. Data are shown as mean values ±standard deviation (SD). Comparison between groups was done using Student's unpaired t-test. Tumor latency was analysed using a Kaplan-Meier survival analysis followed by log rank tests. In all statistical comparisons, P<0.05 was used to indicate a significant difference. Note that for the *ex vivo* and *in vitro* experiments, while tumor cells derived from male and female mice showed similar characteristics in both FVB/N and C57BL/6J backgrounds, only results derived from the tumor cells of female FVB/N mice were shown.
