# Correction: Adiponectin Haploinsufficiency Promotes Mammary Tumor Development in MMTV-PyVT Mice by Modulation of Phosphatase and Tensin Homolog Activities

**DOI:** 10.1371/annotation/14cb7d7e-a921-4ae6-a405-99c60656579f

**Published:** 2009-04-06

**Authors:** Janice B. B. Lam, Kim H. M. Chow, Aimin Xu, Karen S. L. Lam, Jing Liu, Nai-Sum Wong, Randall T. Moon, Peter R. Shepherd, Garth J. S. Cooper, Yu Wang

The following funding information was omitted. RTM's research was supported in part by a breast cancer grant from the US Department of Defense, grant number W81XWH-07-1-0367.

